# Τakotsubo Syndrome After Surgical Removal of the Thyroid Gland and Major Bleeding

**DOI:** 10.7759/cureus.59090

**Published:** 2024-04-26

**Authors:** Charalampos Kakderis, Antonios Kouparanis, Konstantinos C Theodoropoulos, Matthaios Didagelos, Antonios Ziakas

**Affiliations:** 1 Cardiology, Aristotle University of Thessaloniki, AHEPA University Hospital, Thessaloniki, GRC

**Keywords:** left ventriculography, major bleeding, thyroid gland removal, apical ballooning, takotsubo cardiomyopathy (ttc)

## Abstract

A 58-year-old male with a medical history of arterial hypertension, dyslipidemia, and psoriasis was admitted for a scheduled surgical removal of the thyroid gland. During the surgery, the patient suffered severe blood loss caused by vascular complications. After the operation, his electrocardiogram showed diffuse ST segment elevation along with high-sensitivity cardiac troponin T elevation and severe left ventricular systolic dysfunction. An emergency coronary angiography showed unobstructed coronary arteries. However, the left ventriculography demonstrated akinesia of the apical segments and hyperkinesia of the basal segments during systole. The patient was diagnosed with Takotsubo syndrome and he was successfully stabilized over the course of the next few days. Takotsubo cardiomyopathy is characterized by transient left ventricular systolic dysfunction and although the clinical and electrocardiographical presentation is similar to an acute coronary syndrome, the coronary arteries are unobstructed. Stressful events, both physical or psychological, could trigger an excessive catecholaminergic response which can cause the syndrome. Repetitive echocardiograms in our patient demonstrated complete recovery of the systolic function after a few days.

## Introduction

Takotsubo syndrome (TTS) is a reversible systolic dysfunction of the left ventricle which can mimic myocardial infarction. Despite the reduction of the left ventricular ejection fraction (LVEF), the coronary arteries are unobstructed and the wall motion abnormalities of the left ventricle extend beyond the distribution of a single epicardial coronary artery. Takotsubo syndrome was initially described in Japan in 1990 and took its name from a Japanese octopus trap which was similar to the left ventricular apical ballooning silhouette seen in this cardiomyopathy [1]. In Takotsubo cardiomyopathy, severe physical or emotional stress produces ballooning of the left ventricle; it occurs mainly in postmenopausal women [2]. We report a case of Takotsubo syndrome which was caused by vascular complications and severe blood loss during the surgical removal of the thyroid gland.

## Case presentation

**Figure 1:**
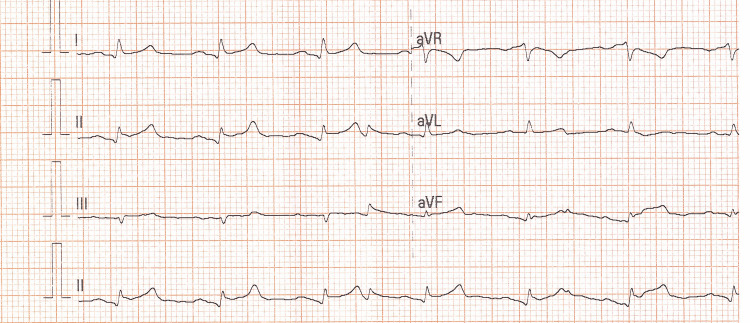
Patient’s electrocardiogram (ECG) with ST-segment elevation in leads I, II, aVF.

**Figure 2:**
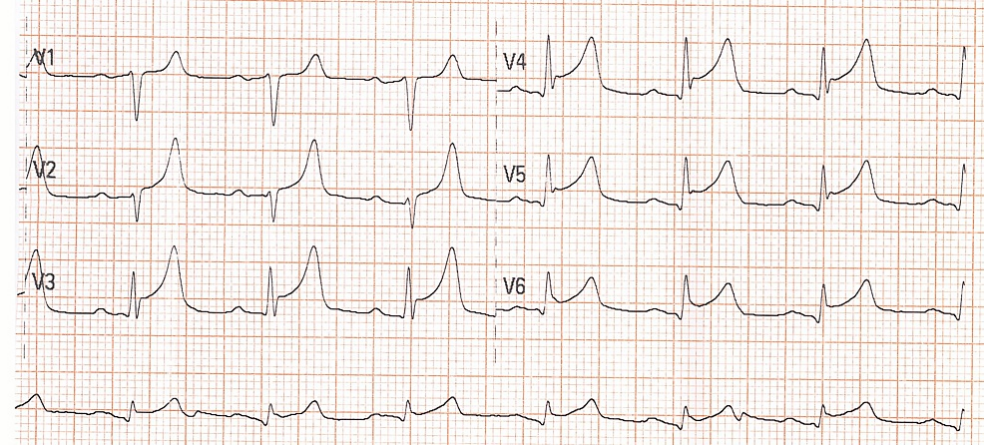
Precordial leads of the patient’s electrocardiogram (ECG) with ST-segment elevation in leads V2-V6.

A 58-year-old male was admitted for a scheduled surgical removal of the thyroid gland. The patient had a medical history of arterial hypertension, dyslipidemia, and psoriasis. He was successfully intubated and transferred to the operating room. During the surgery, the patient suffered severe blood loss due to vascular complications, with a hemoglobin drop from 16.7 to 10.2 g/dL. After the operation, he was transmitted to the surgical intensive care unit (ICU) for further treatment. The patient remained intubated and was administered high doses of inotropes and blood transfusions because of his hemodynamic instability. His electrocardiogram (ECG) after the operation showed acute ST-segment elevation in the leads I, II, aVF, and in the precordial leads V2-V6 (Figures 1, 2).

**Figure 3:**
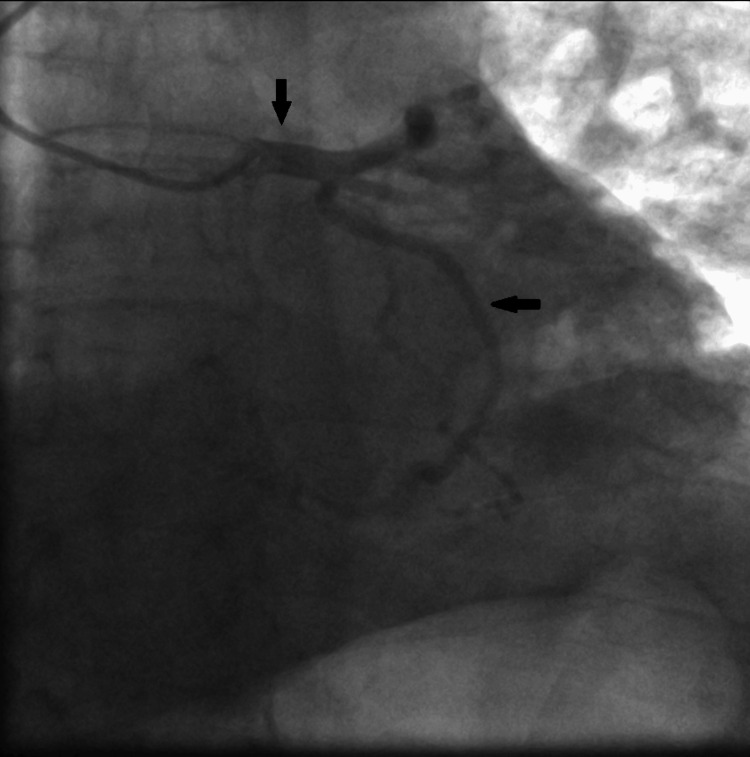
Invasive coronary angiography showing the caudal view in the right anterior oblique (RAO) position The black arrows show the unobstructed left main and left circumflex arteries.

**Figure 4:**
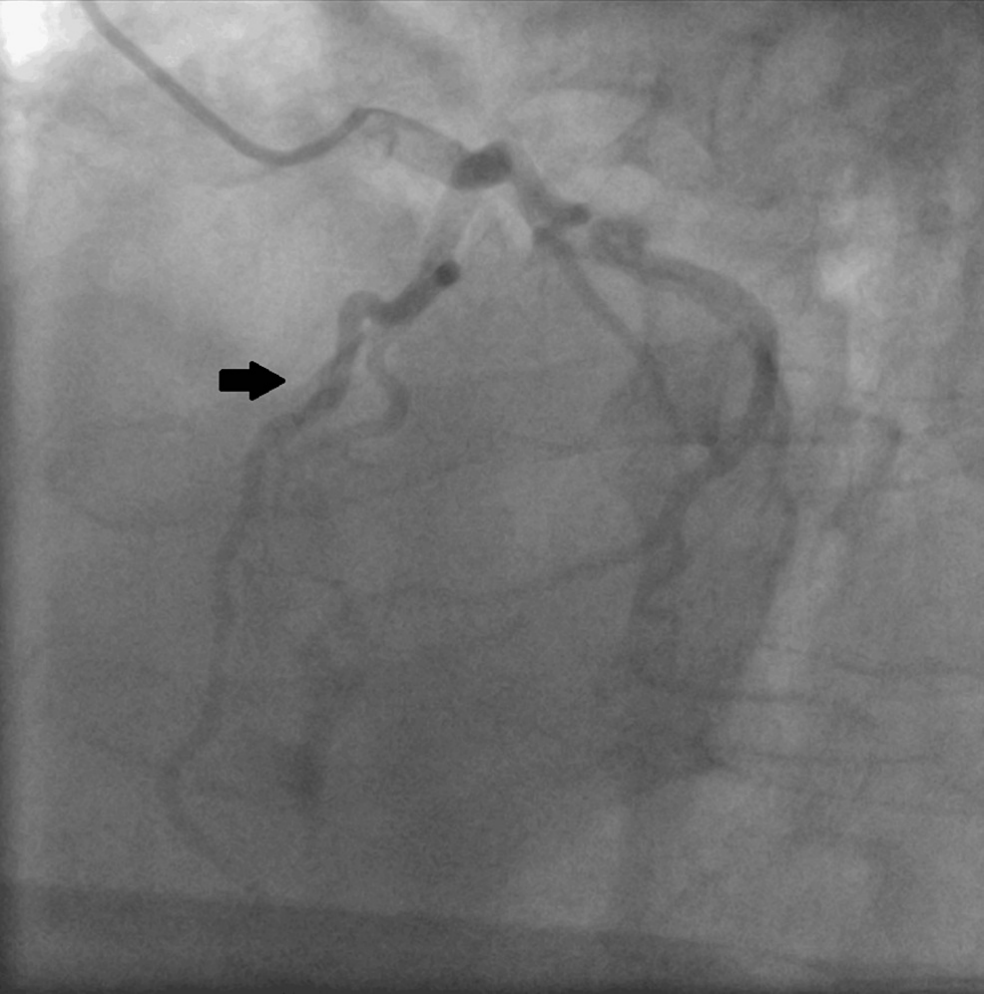
Invasive coronary angiography showing the cranial view in the left anterior oblique (LAO) position The black arrow shows the unobstructed left anterior descending artery.

**Figure 5:**
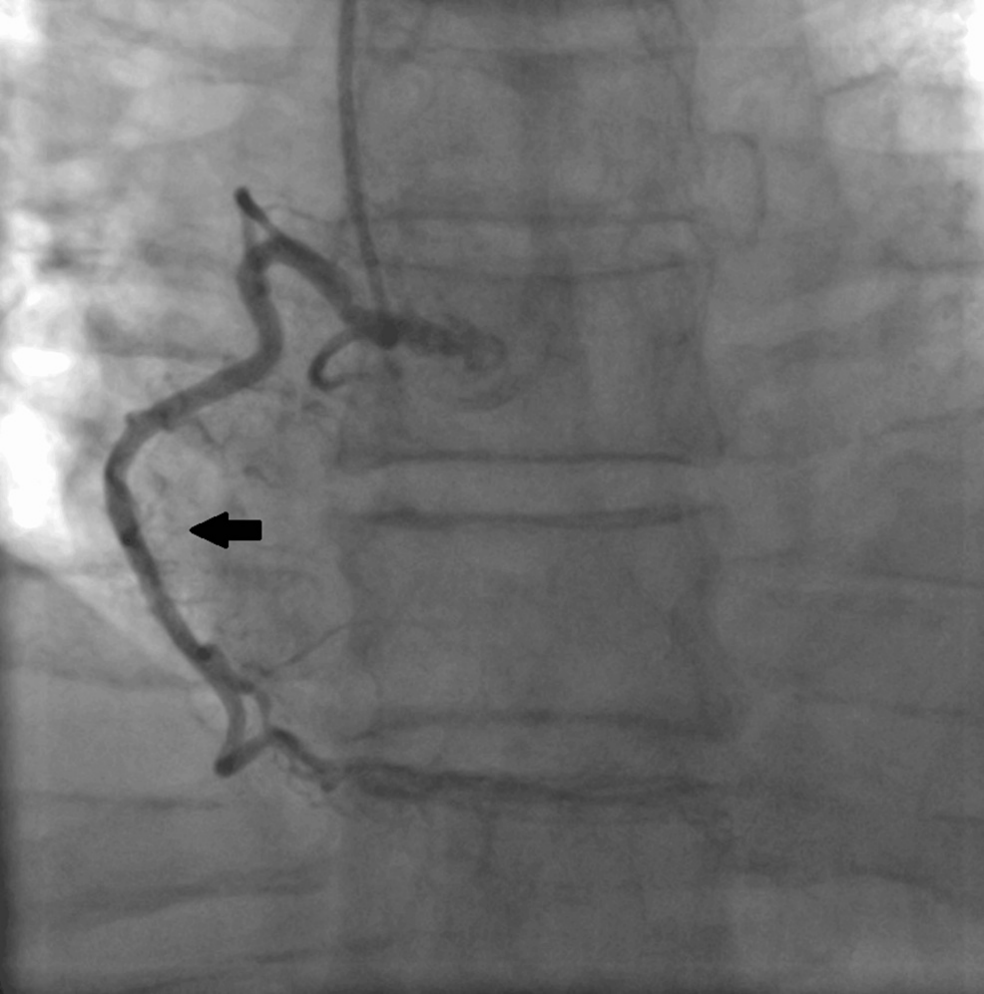
Invasive coronary angiography in the left anterior oblique (LAO) position The black arrow shows the unobstructed right coronary artery.

A bedside transthoracic echocardiogram revealed left ventricular systolic dysfunction with akinesia of the apex and all the apical segments, and left ventricular ejection fraction (LVEF) 35-40%; laboratory findings indicated an elevation of high sensitivity cardiac troponin T to 313 pg/ml (normal range below 14 pg/ml). The patient was immediately transferred to the catheterization laboratory for an emergency coronary angiogram. Coronary angiography was performed via radial access and revealed normal coronary arteries (Figure 3-5).

**Figure 6:**
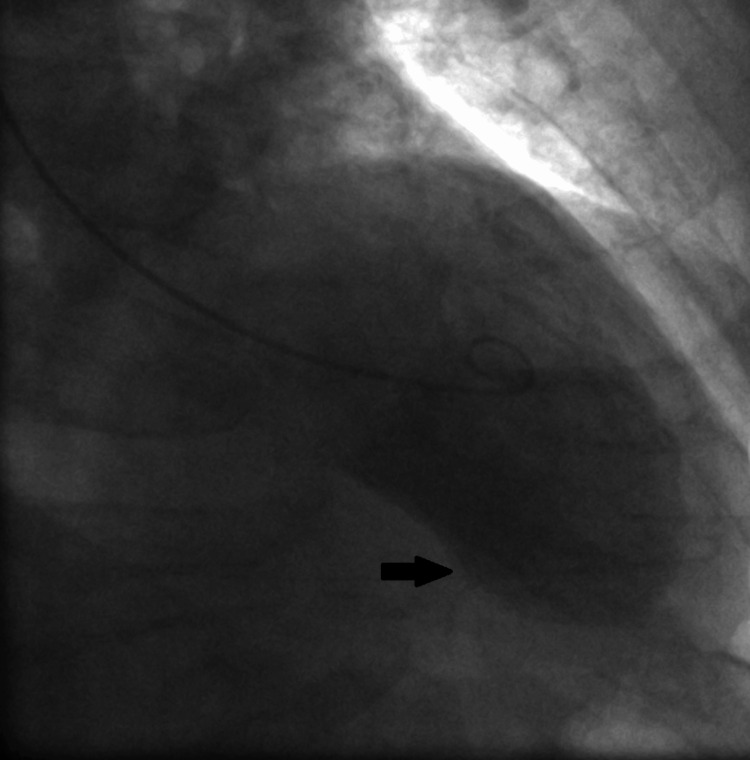
Left ventriculography during diastole (black arrow) in the right anterior oblique (RAO) position

**Figure 7:**
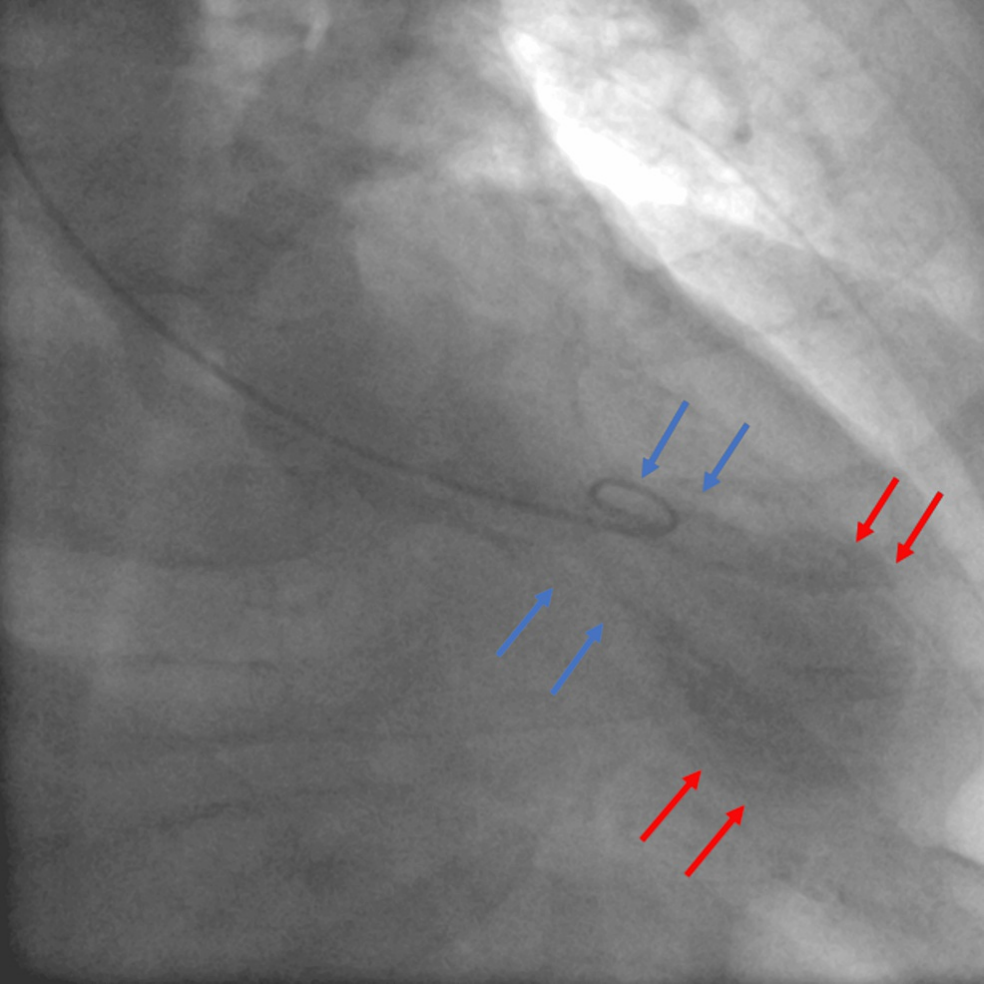
Left ventriculography during systole in the right anterior oblique (RAO) position demonstrating akinesia of the apex and the apical segments of the anterior and the inferior wall (red arrows) with normal systolic function of the mid-ventricular and basal segments (blue arrows).

Left ventriculography demonstrated akinesia of the apex and the apical segments of the anterior and inferior wall along with hyperkinesia of the basal segments during systole (Figure 6, 7).

This presentation was compatible with Takotsubo syndrome probably caused by catecholaminergic stress and severe blood loss during the operation. The patient was stabilized over the course of the next few days, weaned gradually from the inotropic support, and successfully extubated. Repetitive echocardiograms demonstrated complete recovery of the left ventricular systolic function. The patient was on a $\beta$-blocker (bisoprolol 2.5 mg once per day) and angiotensin convertase enzyme (ACE) inhibitor (5 mg perindopril once per day) after his hemodynamic stabilization. The patient made a full recovery and was successfully discharged 16 days after the surgery.

## Discussion

Takotsubo syndrome represents a reversible form of acute heart failure and should always be included in the differential diagnosis of patients with suspected acute coronary syndrome [3]. There are four main anatomical subtypes: apical ballooning (81.7%), midventricular (14.6%), basal (2.2%), and focal (1.5%) [4]. TTS is caused by multiple mechanisms, including excessive sympathetic stimulation, neurogenic stunned myocardium, acute microvascular spasm, and multivessel vasospasm of epicardial coronary arteries. Patients with TTS usually present with chest discomfort and ECG changes which can resemble an acute myocardial infarction (MI). About 2% of the patients suspected to have MI are eventually diagnosed with Takotsubo cardiomyopathy [5]. The revised Mayo Clinic Criteria are used for the diagnosis of the TTS. In order to diagnose the syndrome, patients must have all four criteria, namely transient regional wall motion abnormalities that extend beyond a single epicardial artery distribution, obstructive coronary disease or angiographic evidence of acute plaque rupture should be absent, electrocardiographic abnormalities (such as ST-segment elevation or T-wave inversion) or cardiac troponin rise, and pheochromocytoma or myocarditis have to be excluded [6]. Stable patients are usually treated for the systolic dysfunction with angiotensin-converting enzyme (ACE) inhibitors, beta-blockers, and aldosterone receptor antagonists (MRA); diuretics are used when patients present with pulmonary congestion. The prognosis of Takotsubo syndrome is generally good given that about 95% of the patients will finally recover within the following weeks [5]. However, the in-hospital mortality for TTS is about 5%, which is higher than previously thought [7]. Long-term mortality of TTS patients with atrial fibrillation is significantly higher compared to patients without atrial fibrillation [3].

## Conclusions

In conclusion, Takotsubo cardiomyopathy represents a clinical condition which can mimic myocardial infarction. It is usually caused by severe physical or psychological stress, and should always be included in the differential diagnosis of patients presenting with an acute coronary syndrome.
